# The Effect of Problematic Social Media Use on Happiness among Adolescents: The Mediating Role of Lifestyle Habits

**DOI:** 10.3390/ijerph19052576

**Published:** 2022-02-23

**Authors:** Jiewen Zhang, Claudia Marino, Natale Canale, Lorena Charrier, Giacomo Lazzeri, Paola Nardone, Alessio Vieno

**Affiliations:** 1 Department of Developmental and Social Psychology, University of Padova, 35131 Padova, Italy; claudia.marino@unipd.it (C.M.); natale.canale@unipd.it (N.C.); alessio.vieno@unipd.it (A.V.); 2 Department of Public Health and Paediatrics, School of Medicine, University of Torino, 10126 Torino, Italy; lorena.charrier@unito.it; 3 Department of Molecular and Developmental Medicine, University of Siena, 53100 Siena, Italy; giacomo.lazzeri@unisi.it; 4 National Centre for Disease Prevention and Health Promotion, Italian National Institute of Health, 00161 Roma, Italy; paola.nardone@iss.it

**Keywords:** problematic social media use, happiness, sleep, physical activity, path analysis

## Abstract

Background: Although the relationship between problematic social media use (PSMU) and happiness has been already explored, less is known about the mechanisms that translate PSMU into lower happiness through lifestyle habits. Therefore, the current study focuses on the association between PSMU and happiness, exploring a mediating effect of lifestyle habits (e.g., difficulties in getting to sleep and frequency of physical activity) among Italian adolescents. Methods: A total of 58,976 Italian adolescents (mean age = 13.6, SD = 1.63; age range = 10.5–16.5; 49.4% females) were included. The pattern of relationships specified by the theoretical model was examined through path analysis. Results: Difficulties in getting to sleep (*β* = −0.037, *p* < 0.001) and frequency of physical activity (*β* = −0.012, *p* < 0.001) were mediators in the relationship between PSMU and happiness. Multi-group analyses across gender and age groups (11, 13, and 15 years old) showed that the chain mediating effect of the frequency of physical activity on the difficulties in getting to sleep was not significant for females but significant for males and for all of the three age groups. Overall, females and older ages were sensitive to the whole model. Conclusion: Along with difficulties in getting to sleep and the frequency of physical activity, lifestyle habits may contribute to the association between PSMU and happiness. We also recommend that future studies focus on PSMU in females and older adolescents, as they show more general sleep problems and reduced physical activity.

## 1. Introduction

Social media use could have a double-edged effect on well-being [1]. On the one hand, general users manage lifestyle habits [2], strengthen friendships, obtain social support, and increase social capital, thus increasing self-esteem and well-being through social media [3,4]. On the other hand, social media could be problematic [5]. In particular, problematic social media use (PSMU) is defined as the presence of addiction-like symptoms: preoccupation, tolerance, withdrawal, persistence, displacement, problem, deception, escape and conflict [6]. PSMU can lead to multiple adversities, such as poor academic achievement, cyberbullying, and psychological distress [7,8]. Empirical studies showed that problematic social media users could suffer from lower happiness through negative experiences [9], and/or impaired lifestyle habits of lower physical activity [10] and sleep problems [11].

Although the relationship between PSMU and happiness has been already explored [12], less is known about the mechanisms that translate PSMU into lower happiness through lifestyle habits. Therefore, the current study focuses on the mediating effect of lifestyle habits (i.e., difficulties in getting to sleep and frequency of physical activity) among Italian adolescents.

The association between exposure to social media use and happiness was mixed depending on different feedback or affective experiences [13]. The association between PSMU and well-being was averagely negative [12,14], although Brailovskaia and Schillack [15] pointed out a small positive relation between problematic Facebook use and happiness, but they also explained that social media use for positive experiences also led to PSMU as a result of over-reliance on social media. In fact, more studies pointed out stable negative associations between PSMU and well-being [16,17], that is, positive social reinforcement activated the reward system, leading to problematic use, which decreased psychological well-being. Moreover, there was no direct negative association between PSMU and life satisfaction until the mediating effect of loneliness was included [9]. In summary, despite the mixed results of PSMU being associated with lower happiness, it could be argued that such an association could be explained by other factors. Specifically, an indirect negative association between PSMU and happiness can be hypothesized, while more internal paths need to be dissected, such as the role of competitive activities on happiness [18].

From a neurophysiological perspective, media use through screen devices is related to the release of stress hormones by suppressing melatonin secretion [19,20]. Meanwhile, sedentary behaviors, such as media use, suppress the release of happiness secretions, such as serotonin, noradrenaline, and dopamine [21]. Thus, it is plausible to assume that the results from media use, based on the cited studies, can be cautiously applied to social media use. Furthermore, it is well known that sleep and physical activity are the main determinants that maximize health according to the 24-h movement guidelines for children and adolescents [22,23]. Moreover, from the psychological perspective, activity theory asserts that greater social [24] and leisure [25] activities are related to higher happiness. However, an interplay between activities was observed in influencing happiness: participating in one activity indicated less possibility for another, and these active interactions on happiness were complex [18]. Furthermore, the model of compensatory internet use further explains that only when online activity meets individuals’ psychosocial needs can positive experiences be increased [26]. However, it is crucial to note that activities aiming at satisfying psychosocial needs cannot be premised on impairing the activities of basic needs [27]. From these perspectives, the current study explored the interplay between PSMU and lifestyle habits on happiness. Although online activity was associated with less life satisfaction, the list of specific activities related to happiness is not yet exhaustive [18]. Some studies found that a high frequency of physical activity was positively related to the happiness of adolescents and young people [28,29], and an increase in physical activity prevented addiction-like symptoms of social media use [30]. It can be hypothesized that the frequency of physical activity plays a mediating role in the relationship between PSMU and happiness among adolescents.

In addition, sleep, as another daily basic need, seems to be a link between PSMU and happiness. Prior research showed negative relationships between problematic internet use and sleep quality, as well as life quality [31]. More precisely, a higher level of sleep problems was directly associated with lower happiness [32]. It follows that PSMU can be directly related to a series of health complaints, including having difficulties falling asleep [33]. However, as for why these variables affect each other, Marino and Vieno [34] indicated that sleep plays a mediating role between computer use and happiness. Then, Vernon and Modecki [35] further clarified that sleep disruptions mediate the relationship between PSMU and ill-being among adolescents.

However, to our knowledge, only one study explored the correlations and mediations between technology use, sleep, physical activity, and mental health as follows: the mediating role of sleep time and physical activity between social media use and overall mental health [10]. It is surprising that no study combined the four variables (PSMU, sleep, physical activity, and happiness) together in an integrated model to analyze the internal competition mechanism, even though PSMU, recognized as an addiction-like symptom activity [6], has a higher negative impact on happiness than general social media users [16]. Briefly, PSMU increases difficulties in getting to sleep [33,35], which is related to low happiness [32,34]; PSMU decreases the frequency of physical activity [30,36], which is related to low happiness [28,29]. In addition, evidence from a meta-analytic review exhibited that physical activity has a beneficial effect on sleep [37], so a chain mediation path of the frequency of physical activity to difficulties in getting to sleep was also explored in the hypothesized model.

The current study focuses on the pathways of PSMU on happiness. We hypothesized a negative association between PSMU and happiness (H1). Moreover, according to the activity theory [24], need theory [27] and the 24-h movement guidelines framework [22,23], we tested the mediating role of lifestyle habits. The hypothesized pathways are as follows: the higher the levels of PSMU, the lower the frequency of physical activity (H2) and the higher the frequency of physical activity, the more happiness (H3). The higher the PSMU levels, the greater the difficulty in getting to sleep (H4), and the greater the difficulty in getting to sleep, the lower the happiness (H5). The higher the frequency of physical activity, the lesser the difficulty in getting to sleep (H6). Frequency of physical activity and difficulties in getting to sleep are mediators in the relationship between PSMU and happiness (H7).

**Figure 1:**
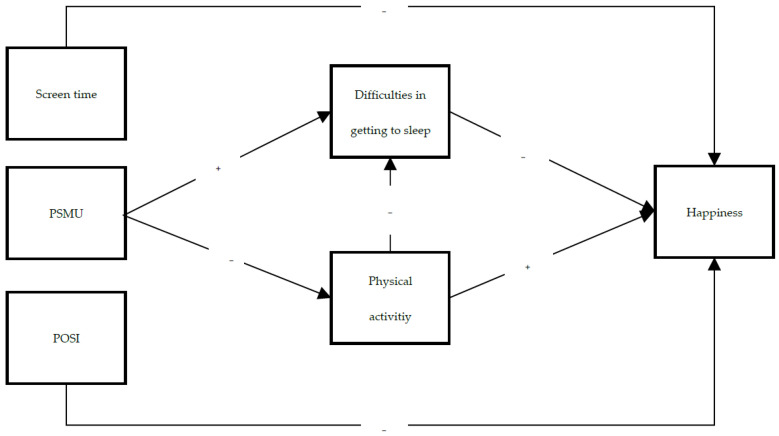
Hypothesized model. The symbols for the expected relationships (“+” in the positive and “−” in the negative); POSI = preference for online social interaction, PSMU = problematic social media use.

Additionally, the relationship between PSMU, difficulties in getting to sleep, frequency of physical activity and happiness by gender and age seems to still be debated. In particular, PSMU is negatively related to mental health, regardless of gender and age [38], while higher frequency of social media use indicates more health problems in older age, as well as in the female adolescent group [39]. Accordingly, the groups of boys and girls, as well as the groups of early, middle and late adolescents may differ in the relationships between PSMU, difficulties in getting to sleep, frequency of physical activity and happiness. Therefore, the two groups across gender and the three groups across age were analyzed separately to explore different pathway effects. Finally, preference for online social interaction (POSI) and screen time are linked to PSMU and ill-being [40,41,42,43,44]. Thus, POSI and screen time were included as covariates of happiness (see Figure 1).

## 2. Methods

### 2.1. Participants and Procedure

Data were gathered from the Health Behavior in School-aged Children (HBSC) survey. HBSC is a cross-national survey hosted by the World Health Organization (WHO), conducted every 4 years in about 50 countries. The aim is to monitor school-aged children’s health. In particular, the HBSC survey has been recognized as the only national surveillance by Italian Legislative Decree since 2017 and is involved in the Italian National Institute of Health as a coordinator [45]. In the current study, 2018 Italian HBSC data including 11-, 13-, and 15-year-old adolescents corresponding to the junior 1, junior 3 and senior 2 grades were used. An international protocol that outlines standard sampling procedures, data coding and processing methods was compiled [46]. Adolescents participated in the Italian survey anonymously and voluntarily. In this study, the following variables were chosen: PSMU, difficulties in getting to sleep, frequency of physical activity, happiness, screen time and POSI. A total of 58,976 adolescents aged from 10.5 to 16.5 years by three grades (mean age = 13.6, SD = 1.63; 49.4% females; 34.1% with 11-year-olds, 35.4% with 13-year-olds) were included.

### 2.2. Measures

Problematic social media use. PSMU was assessed with the Social Media Disorder Scale [6]. It measured nine symptoms (i.e., preoccupation, tolerance, withdrawal, persistence, displacement, problem, deception, escape and conflict) of PSMU with nine items. A sample item is “During the past year, have you regularly found that you can’t think of anything else but the moment that you will be able to use social media again?” (preoccupation). Participants were asked to think about the last year and answer “yes” or “no” to each item. Responses were summed to obtain a continuous PSMU score so that higher total scores indicated higher PSMU. In this study, the Cronbach’s alpha was 0.72 (95% CI = 0.717–0.724). Positive correlation between PSMU and POSI was 0.3, which reflected moderate criterion validity. The standardized factor load of PSMU was higher than 0.5, which indicated acceptable construct validity.

Difficulties in getting to sleep. An item of the Health Complaint Scales was included in HBSC Symptom Checklist [47]. It measured the difficulties of adolescents in getting to sleep by asking: “In the last 6 months: how often have you had difficulties in getting to sleep?” Answers ranged from 1 (rarely or never) to 5 (about every day). Higher score indicated more difficulties in getting to sleep in this study.

Frequency of physical activity. A single item adapted from the Physical Activity Screening Measure was used to assess the frequency of physical activity [48]. Respondents answered the following question: “Over the past 7 days, on how many days were you physically active for a total of at least 60 min per day?”. Response options ranged from 1 (1 day) to 7 (7 days). A higher score indicated a higher frequency of physical activity.

Happiness. Happiness was measured with the Cantril’s Ladder of Life Scale [49]. Participants were asked to answer the following question: “In general, where on the ladder do you feel you stand at the moment?” from 0 (worst possible life) to 10 (best possible life). Higher score represented higher happiness.

Controls. Screen time and POSI were included as control variables. A single item was used to measure screen time related to watching TV/DVD/VIDEO on weekdays. Answers ranged from 1 (none at all) to 9 (about 7 or more hours a day). Higher scores indicated intense screen time. POSI was a subscale of the Italian version of the Generalized Problematic Internet Use Scale 2 [50]. It comprised 3 items assessing preference for online social interaction (e.g., “On the internet, I talk more easily about secrets than in a face-to-face encounter”) rated on a 5-point scale from 1 (strongly disagree) to 5 (strongly agree). Higher total scores indicated higher POSI. In this study, the Cronbach’s alpha was 0.90.

### 2.3. Statistical Analysis

First, the preliminary analysis included descriptives, Pearson’s correlations among study variables, and a series of one-way ANOVAs were run to explore possible differences by gender and age groups in the variables of interest. Second, a path analysis was run with a robust maximum likelihood estimator (MLR) [51] for observed variables; indirect paths from the independent variable to the dependent variable via mediators were tested using the product of coefficients approach [52,53]. In the hypothesized theoretical model (Figure 1), the independent variable was PSMU. Mediators were frequency of physical activity and difficulties in getting to sleep. The outcome variable was happiness. Control variables were screen time and POSI. Third, in order to explore whether the hypothesized model would differ across gender and age groups, a series of multi-group path analyses were run. R-square and effect sizes were considered to evaluate the goodness of the whole model and its paths, because without latent variables, standard fit indices (e.g., CFI, χ^2^) were not particularly useful since they are often not sensitive to the errors in model equations that are expressed from the Ψ matrix [54]. Data analyses were performed using Mplus. 8.0 and SPSS version. 24.

## 3. Results

### 3.1. Preliminary Analysis

First, 0.74% of the sample had missing data on at least one variable, with PSMU having the most missing data (9.6% of respondents), which were smartly excluded from the analyses. A total of 8.95% of the adolescents answered “yes” to at least 6 items of the Social Media Disorder Scale and were classified as problematic social media users. More than 12.49% of adolescents had difficulties in getting to sleep about every day. Nearly 16.60% of adolescents engaged in physical activity only 1 day or never in the past 7 days. Happiness turned out to be lower than the middle level for 5.8% of the adolescents. Second, means, standard deviations and Pearson’s correlations between variables are presented in Table 1, while the one-way ANOVAs analysis is shown in Table 2. As expected, PSMU was negatively associated with happiness and frequency of physical activity, while positively related to difficulties in getting to sleep. There was a strong negative correlation between difficulties in getting to sleep and happiness, while there was a positive correlation between frequency of physical activity and happiness. However, PSMU, frequency of physical activity, difficulties in getting to sleep and happiness differed significantly across gender and grades. Specifically, females’ PSMU and difficulties in getting to sleep were higher than males’, whereas males’ frequency of physical activity and happiness were higher than females’. Meanwhile, the lowest PSMU and difficulties in getting to sleep were observed in the 11-year-olds group that showed the highest frequency of physical activity and happiness. This means that older age adolescents had significantly higher PSMU and difficulties in getting to sleep, with lower frequency of physical activity and happiness, compared to the 11-year-olds group.

### 3.2. PSMU on Happiness

**Figure 2:**
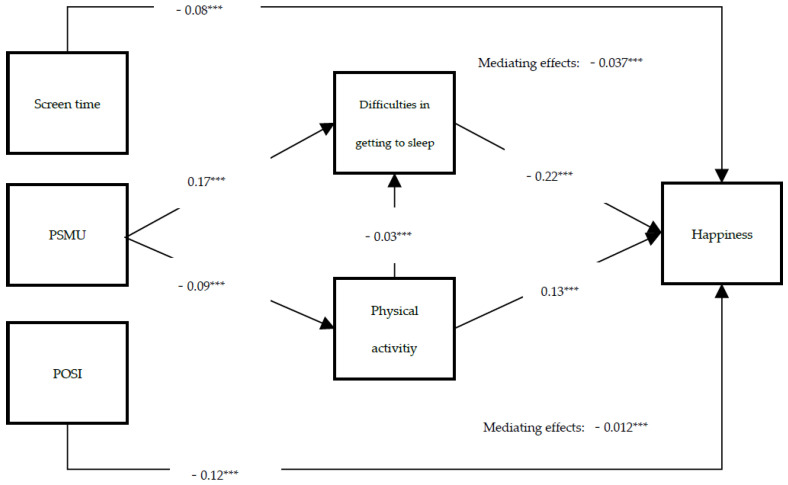
Path diagram of the structural equation model 0 with significant pathways. All coefficients are standardized. POSI = preference for online social interaction, PSMU = problematic social media use. The above mediating effect refers to difficulties in getting to sleep while the bottom refers to physical activity. *** *p* < 0.001.

The results of the theoretical model are shown in Figure 2. The significance level of the path coefficients was 0.001. All direct paths were significant. In particular, PSMU was positively associated with difficulties in getting to sleep while being negatively associated with the frequency of physical activity. On the other hand, difficulties in getting to sleep were negatively associated with happiness, whereas the frequency of physical activity was positively associated with happiness. There was also a weak but significant association between the frequency of physical activity and the difficulties in getting to sleep. The control variables, screen time and POSI were negatively associated with happiness in the model. In terms of indirect paths, the product of coefficients approach supported two mediators between PSMU and happiness. Moreover, the chained mediating effect from the frequency of physical activity to difficulties in getting to sleep was negative. PSMU is associated with less happiness in adolescents by affecting their lifestyle habits of frequency of physical activity and difficulties getting to sleep; PSMU had more than three times the influence on happiness through sleep than through physical activity. Regarding the fit of the model, the whole model explained 9.6% of the total variance in happiness.

### 3.3. Gender Differences

**Figure 3:**
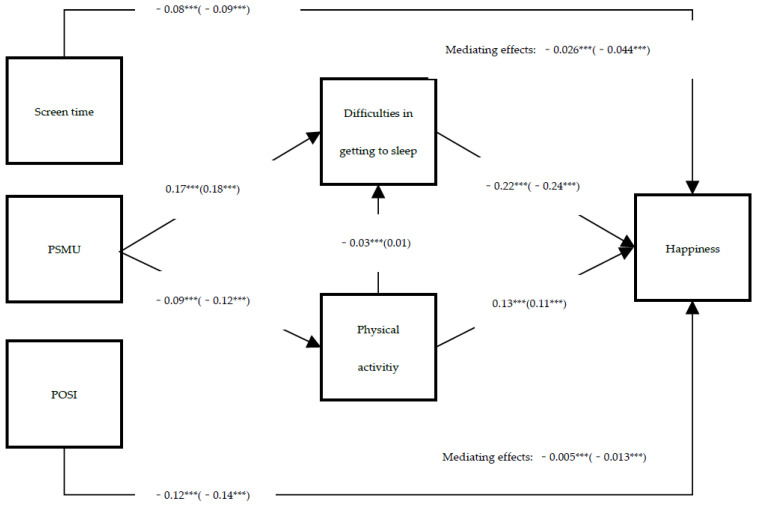
Path diagram of the structural equation model 1 with significant pathways. All coefficients are standardized. Females of model 2 is in brackets. POSI = preference for online social interaction, PSMU = problematic social media use. *** *p* < 0.001. The above mediating effect refers to difficulties in getting to sleep, while the bottom refers to physical activity.

The results of the tested model across gender are shown in Figure 3. The direct paths of the model were significantly (at least *p* < 0.001) different by gender. Specifically, PSMU was more sensitive to the frequency of physical activity of females. However, the frequency of physical activity had a greater effect on difficulties in getting to sleep and happiness for males than females. Regarding indirect relationships, the mediating effect of difficulties in getting to sleep was significantly greater than that of frequency of physical activity for females than males. The chained mediating effect via frequency of physical activity and difficulties in getting to sleep was only significant for males. In general, females were more sensitive to the effect of PSMU on happiness via the two mediators than males. The whole model explained 11% of the total variance on happiness for females than that for males of 7.3%.

### 3.4. Age Differences

**Figure 4:**
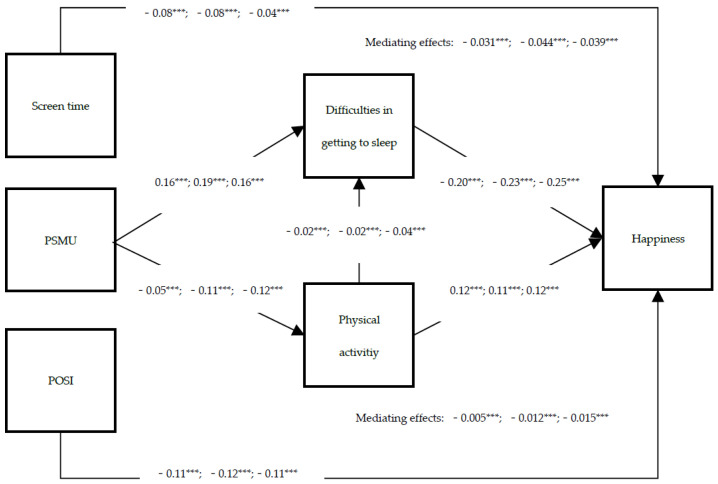
Path diagram of structural equation model 2 with significant pathways. All coefficients are standardized. The estimates from left to right are 11-year-olds of model 3, 13-year-olds of model 4 and 15-year-olds of model 5. POSI = preference for online social interaction, PSMU = problematic social media use. The above mediating effect refers to difficulties in getting to sleep, while the bottom refers to physical activity. *** *p* < 0.001.

The results of the tested model across age groups are shown in Figure 4. The direct paths of the model differed by age groups. We observed that PSMU had apparently the lowest negative impact on frequency of physical activity in the 11-year-old group, compared to the other two age groups. PSMU had a relatively low negative impact on difficulties in getting to sleep in the 11-year-old group. At the same time, this group suffered least from the effect of difficulties in getting to sleep on happiness. However, the frequency of physical activity on happiness had a similar positive effect, no matter the age group. Regarding indirect paths, the mediating effect of difficulties in getting to sleep was greater than that of the frequency of physical activity, no matter the age group. The chained mediating effect via the frequency of physical activity and difficulties in getting to sleep was significant for all of three age groups but highest in the 15-year-old group. Overall, the smallest age group suffered less from the effects of PSMU on happiness via the two mediators. The whole model explained 7.6% of the total variance on happiness in the 11-year-old group, and the other two groups both explained 9.7% of the total variance on happiness.

## 4. Discussion

Overall, the study confirmed that lifestyle habits contribute to explaining the association between PSMU and happiness among adolescents. This study expanded the current literature on mechanisms that mainly focus on personal psychological risk factors, such as self-esteem [55], enriching the current knowledge of the crucial role of lifestyle habits.

It is meaningful that the associations of PSMU with the two habits (i.e., difficulties in getting to sleep and frequency of physical activity) had a reverse effect on happiness. On the one hand, PSMU was positively related to difficulties in getting to sleep. When adolescents showed problematic patterns of social media use, they favored a single-dimension life, consequently spending less time and less energy in other important real-life activities [56]. Moreover, this finding extends the evidence for the significant associations between technology use and sleep problems. Although technology use of video gaming, phone, computer and internet use and television were neither risk factors nor protective factors for sleep [57], PSMU could be a risk for sleep. For instance, it is clear that the higher the level of PSMU, the more the adolescents felt difficulties in getting to sleep. In turn, difficulties in getting to sleep decreased happiness.

On the other hand, PSMU was negatively associated with the frequency of physical activity. This means that PSMU leads to a lower frequency of physical activity. The time competition effect might help explain this result, exhibiting that the sedentary pattern of PSMU was responsible for low physical activity [58]. Then, PSMU indirectly reduced happiness among adolescents. It is true that a high frequency of physical activity was associated with perceived positive emotion and offline social care [30,59]. Again, these findings confirmed the time competition, indicating the interplay of online leisure activity and basic-needs activity in real life, which revealed the effect of lifestyle habits, due to PSMU [18], which decreased happiness among adolescents.

Furthermore, except for the separate mediating effects between PSMU and happiness, there is the chained mediating effect from PSMU via both the frequency of physical activity and difficulties in getting to sleep on happiness. It means that PSMU, along with inactive physical activity, suppressed the positive emotions of a high frequency of physical activity, while releasing more negative emotions [60], such as perceiving stress to disturb stable sleep activity [20]; then, the poor regulation of circadian rhythms led to difficulties in getting to sleep, reducing happiness among adolescents [32]. On the other hand, another, further explanation for the whole phenomenon is in the use of online activities to seek satisfaction, while focusing on online activities led to ignore offline lifestyle habits; then, finally, this process related to lower happiness [26,27].

In addition, POSI and screen time were both significantly associated with less happiness in the current study. These results were consistent with the recent proposals that more hours of screen activities are related to lower psychological well-being due to the reduction in face-to-face social interaction [41], or due to the possibility of adolescents’ reduced contact with nature [61]. POSI was also negatively associated with psychological well-being in a study about the relationship between problematic internet use and psychological well-being [62].

We also found that PSMU had higher negative consequences among females than among males. It means that females spent less time engaged in physical activity and experienced more difficulties in getting to sleep and less happiness. A possible explanation for this result may refer to the uses and gratifications theory of online media, in which media users seek gratification based on individual needs [63]. Previous studies also indicated that females were more sensitive to the function of interpersonal interactions on social media to seek interpersonal gratification [64,65,66]. The current study provided more evidence that females are more likely to be affected by PSMU than males, in terms of higher level of PSMU and less healthy lifestyle habits. However, the benefit of physical activity on sleep was not significant among female adolescents, while difficulties in getting to sleep among males was probably affected by the frequency of physical activity. Existing evidence suggesting that psychological stimulation plays a mediating effect between screen media use and insufficient sleep among youths [67], might help to explain this result. In other words, other psychological factors may likely affect the relationship between PSMU and difficulties in getting to sleep beyond frequency of physical activity, and then decrease happiness among female adolescents. Future research could focus on girls’ psychological factors to explore the relationship between PSMU and sleep to affect happiness.

In the present study, frequency of physical activity and difficulties in getting to sleep played a mediating role and chain mediating role to significantly explain the relationships between PSMU and happiness, regardless of age groups among adolescents. Additionally, older adolescents showed higher levels of PSMU and worse lifestyle habits than lower age adolescents. These findings first confirmed the stable associations between PSMU, lifestyle habits and happiness among different ages, in line with previous studies [39,68], although there were subtle differences in effect sizes for these associations. Despite the limited evidence, another longitudinal study reported that spending more time on screen media use in the early life stage resulted in engaging in a lower frequency of physical activity in the later life stage due to time displacement, but there was an insignificant association between screen media time and sleep duration [69]. The current study extends the literature on difficulties in getting to sleep significantly mediating the association between PSMU and happiness and provides support for understanding PSMU patterns to disturb regular sleep habits. More interestingly, problematic social media users were more likely to suffer from difficulties in getting to sleep, rather than a lower frequency of physical activity, decreasing happiness. These findings suggest that impaired lifestyle habits, especially sleep, could have negative effects on happiness among adolescents, especially among older age group. However, more longitudinal studies should verify this in the future.

Several limitations of the current study should be acknowledged. Firstly, the current study is cross sectional and uses a path analysis, which is only suggestive of causal effects. Future studies should use a longitudinal design to replicate the proposed mechanisms between PSMU, lifestyle habits, and happiness. Secondly, even though difficulties in getting to sleep and frequency of physical activity were the most important daily activity habits for mental health [70], there are certainly other lifestyle habits that were not considered in the current model. For example, prior research indicated that excessive social media use disrupted dietary habits [71], and poor diet is related to major depression [21]. Therefore, whether dietary habits can be a mediator between PSMU and happiness needs to be further explored in the future. Thirdly, the current study reflected small effects in our model; even though it made sense when considered at scale and over time [72], there may be other internal influencing mechanisms that are more important and need to be explored in the future (such as specific sleep problem types). Fourthly, the assessment of physical activity and happiness is less robust because single items were used in the current study. For physical activity, measurement of the type and intensity of the activity, as well as the total time spent on physical activity per week, is lacking. For happiness, the subjective satisfaction with life was assessed, but there is a lack of information about other aspects of happiness and well-being.

In conclusion, this study found that lifestyle habits (difficulties in getting to sleep and frequency of physical activity) mediated the relationship between PSMU and happiness among adolescents. A potential theoretical mechanism was suggested from problematic technology use (PSMU) to well-being (happiness). In addition, the possibility of this mechanism was verified in different gender and age groups. Specifically, females and older adolescents were more sensitive to this model. It is important to explore strategies for improving well-being among adolescents, especially in educational programs or interventions aimed at increasing physical and mental health and preventing PSMU among adolescents. Indeed, the results suggested the importance of lifestyle habits and highlighted their relationships with PSMU. In addition, the current model might be helpful to further improve the awareness of parents, schools, and government education departments to regulate adolescents’ social media use, which may prevent or improve on the physical and mental distresses caused by PSMU in the future.

## Figures and Tables

**Table 1:** Descriptive statistics and Pearson correlation analyses (PSMU = problematic social media use); POSI = preference for online social interaction, ** *p* < 0.01. M = mean.

	M	SD	*n*	1	2	3	4	5
1. PSMU	2.32	2.16	53,292	1	-	-	-	-
2. Frequency of physical activity	3.42	1.98	58,516	−0.09 **	1	-	-	-
3. Difficulties in getting to sleep	2.22	1.47	58,539	0.17 **	0.04 **	1	-	-
4. Happiness	7.58	1.78	58,462	−0.20 **	0.15 **	−0.24 **	1	-
5. POSI	5.79	3.17	55,916	0.30 **	−0.04 **	0.11 **	−0.16 **	1
6. Screen time	1.83	1.51	57,638	0.18 **	−0.08 **	0.07 **	−0.12 **	0.12 **

**Table 2:** Between group differences in problematic social media use (PSMU), frequency of physical activity, difficulties in getting to sleep and happiness. M = mean.

Variables	PSMU			Frequency of Physical Activity			Difficulties in Getting to Sleep			Happiness		
	M (SD)	*p*	η2	M (SD)	*p*	η2	M (SD)	*p*	η2	M (SD)	*p*	η2
Male	2.11 (2.12)	0.000	0.009	3.76 (1.98)	0.000	0.031	2.07 (1.40)	0.000	0.012	7.73 (1.70)	0.000	0.007
Female	2.52 (2.19)			3.06 (1.92)			2.38 (1.52)			7.43 (1.84)		
11-year -olds	2.17 (2.20)	0.000	0.004	3.70 (1.96)	0.000	0.016	2.21 (1.49)	0.022	0.000	7.99 (1.80)	0.000	0.036
13-year -olds	2.50 (2.17)			3.44 (1.96)			2.21 (1.46)			7.55 (1.74)		
15-year -olds	2.25 (2.09)			3.08 (1.99)			2.25 (1.45)			7.16 (1.69)		

## Data Availability

The data presented in this study are available on request from the corresponding author. The data are not currently publicly available because the international datafile is restricted for the use of HBSC member country teams for a period of three years from its completion; thereafter it will be open access. https://www.uib.no/en/hbscdata/113290/open-access (accessed on 21 January 2022).
